# Metacognitive accuracy differences in Parkinson’s disease and REM sleep behavioral disorder relative to healthy controls

**DOI:** 10.3389/fneur.2024.1399313

**Published:** 2024-05-27

**Authors:** Maria Bălăeţ, Falah Alhajraf, Niall J. Bourke, Jessica Welch, Jamil Razzaque, Paresh Malhotra, Michele T. Hu, Adam Hampshire

**Affiliations:** ^1^Department of Brain Sciences, Imperial College London, London, United Kingdom; ^2^Oxford Parkinson’s Disease Centre, Nuffield Department Clinical Neurosciences, University of Oxford, Oxford, United Kingdom; ^3^Centre for Neuroimaging Sciences, Institute of Psychiatry, Psychology and Neuroscience, King’s College London, London, United Kingdom

**Keywords:** Parkinson’s disease, REM sleep behavioral disorder, metacognition, metacognitive accuracy, online cognitive assessments

## Abstract

**Background:**

Metacognition is the ability to monitor and self-assess cognitive performance. It can be impaired in neurodegenerative diseases, with implications for daily function, and the ability of patients to reliably report their symptoms to health professionals. However, metacognition has not been systematically assessed in early-mid stage Parkinson’s disease (PD) and REM sleep behavioral disorder (RBD), a prodrome of PD.

**Objectives:**

This study aimed to evaluate metacognitive accuracy and self-confidence in PD and RBD patients across various cognitive tasks.

**Methods:**

We conducted detailed computerized cognitive assessments with 19 cognitive tasks within an established PD and RBD cohort. Participants self-rated their performance post-task. Metacognitive accuracy was calculated by comparing these ratings against objective performance and further analyzed against clinical and mental health factors.

**Results:**

PD and RBD patients’ metacognitive accuracy aligned with control subjects. However, they exhibited lower confidence across cognitive domains, reflecting their reduced cognitive performance. A notable inverse correlation was observed between their confidence and MDS-UPDRS I and II scales and HADS anxiety and depression scores.

**Conclusion:**

Our findings indicate that patients with early to mid-stage PD and RBD are generally aware of their cognitive status, differing from other neurological disorders. The inverse relationship between patient confidence and symptoms of depression, anxiety, and daily life challenges underscores the impact of emotional and functional difficulties on their self-perception of cognitive abilities. This insight could be significant for understanding how these conditions affect mental health, aiding clinicians in developing more effective patient care strategies.

## Introduction

Metacognition refers to the ability to self-reflect on one’s own cognitive processes. Accurately building an image of oneself is key to human activity behavior regulation (1) and directly informs decision-making, problem-solving and general well-being (2, 3). The construct of metacognitive accuracy, which measures the congruence between one’s judgments of their cognitive performance and actual task performance, has garnered significant interest in psychological research. Good metacognitive accuracy, where there is a close correspondence between predicted and actual performance, is indicative of well-calibrated self-assessment abilities. Measures of metacognitive accuracy, derived from task performance, have been shown to be critical indicators of the functional integrity of an individual’s metacognitive processes (4).

Although aging in itself is not generally detrimental to metacognitive accuracy, which remains stable across most cognitive domains (5), it is important to note that progressive neurodegenerative diseases leading to dementia frequently exhibit a decline in metacognitive capabilities (6). Past research has established a connection between damage to the anterior prefrontal cortex and reductions in metacognitive accuracy (7). Moreover, in conditions such as Parkinson’s disease (PD), there is observed degeneration in the frontal lobes (8), which has been associated with cognitive decline (9). Given that the patterns of metacognitive impairment observed post brain injury overlap with those seen in the cognitive impairments characteristic of PD, this presents a compelling rationale for more detailed investigations into the potential impact of PD on metacognitive accuracy.

PD is a progressive neurodegenerative disease characterized by both motor and nonmotor symptoms, including cognitive deficits that can manifest as PD-dementia, affecting patient independence and increasing caregiver burden (10–12). REM sleep behavior disorder (RBD) is an early PD indicator, with a 1% incidence in the elderly, similar to PD (13). RBD carries an uneven risk of progressing to PD or related diseases – about 30% within three years, with higher risks (up to 65%) for certain groups, and 73.5% after 12 years (14, 15). While profound disturbances in self-awareness are not typical in PD, emerging evidence suggests potential impacts on key metacognitive aspects (6).

Research into metacognition within PD is notably scant, particularly concerning its early stages. Additionally, the current body of literature overlooks metacognition in the context of RBD. Some investigations have indicated preserved aspects of metacognition in PD; for instance, research by Oh-Lee et al. (16) suggests that PD patients may not suffer deficits in specific facets of metamemory, such as the tip-of-the-tongue phenomenon. Conversely, there are studies indicating compromised self-awareness and metacognitive functions in PD. For example, there is some evidence to suggest that patients with PD, especially those in advanced stages of cognitive decline, often exhibit anosognosia. This condition, which is characterized by a lack of awareness of one’s own illness, is believed to stem from degeneration in brain areas responsible for self-perception. Notably, this lack of awareness in PD patients was noted to be inversely related to depression (17). Studies have also identified impairments in olfactory metacognition in PD, with patients demonstrating a reduced ability to accurately assess their own proficiency in smell identification (18). Additionally, self-awareness of impulse-control disorders appears to be either comparable or heightened in PD patients with impulse-control disorders when contrasted with those without (19). In studies where PD patients with comorbid gambling disorder were assessed using the Iowa gambling task followed by metacognitive self-reports, metacognitive abilities seemed compromised only in the presence of both conditions, suggesting that poor impulse control may act as a conduit for metacognitive impairment in PD (20).

Mental health status is likely to play a role in biasing metacognitive judgments (although this is unlikely to be specific to neurodegenerative conditions). Symptoms of depression and anxiety, which often manifest as worry and rumination, have been shown to influence metacognitive assessments in both healthy individuals and PD patients, who frequently contend with these issues (21). A maladaptive metacognitive style has indeed been associated with higher levels of distress in PD patients (22).

What the current general literature lacks is a comprehensive examination of metacognition, particularly metacognitive accuracy derived from task performance across various cognitive domains. Such an investigation would provide an objective measure that contributes to our understanding of higher-order metacognitive processes. There is a need to ascertain the extent of impairment in metacognition within PD, evaluate whether similar or different levels of deficits are present in RBD, and understand how these cognitive aspects relate to the symptoms of anxiety, depression, and the clinical manifestations experienced by patients.

Our study explored self-reported confidence and metacognitive accuracy in relation to Hospital Anxiety and Depression Scale (HADS) and Movement Disorder Society-Sponsored Revision of the Unified Parkinson’s Disease Rating Scale (MDS-UPDRS) scales in PD and RBD patients within the Discovery Cohort (23). It aimed to identify whether metacognitive processes are affected in early and prodromal PD stages, potentially serving as early indicators of the disease. Participants completed 19 cognitive tasks assessing executive function, reasoning, attention, memory, language, and motor function (24), followed by performance ratings from 1 (low confidence) to 100 (high confidence). Metacognitive accuracy was calculated by comparing these ratings with actual performance (25). The hypothesis was that PD and RBD patients would show impaired metacognitive accuracy and lower confidence compared to controls, correlating with anxiety, depression, and symptom severity as per MDS-UPDRS I, II, and III scales.

## Methodology

### Participants recruitment

Individuals from the Oxford Discovery Cohort (23), enrolled within three and a half years from their initial diagnosis during 2010–2016, were regularly evaluated using clinical scales, including the Montreal Cognitive Assessment (MoCA). From 2020 to 2022, those scoring above 24 on their latest MoCA (indicating no mild cognitive impairment) were invited for computerized cognitive tasks on Cognitron.^1^ The study comprised 59 PD patients, 54 with isolated RBD, and 50 controls, with 56 PD, 50 RBD, and 46 controls completing all tasks. Sociodemographic and clinical data at baseline and most recent assessment pre-cognitive tests are reported in Supplementary Table S1.

Ethical approval was given by the South Central-Oxford A Research Ethics Committee in accordance with the Declaration of Helsinki 1964, Ethics Ref: 16/SC/0108. All participants provided informed consent prior to completing the survey.

### Discovery cohort patient selection

Participants with a diagnosis of PD or RBD were included in the Oxford Discovery Cohort (23). Participants with PD were diagnosed based on the UK PD Brain Bank Criteria (26). The diagnosis of RBD was made on the basis of polysomnographic evidence according to International Classification of Sleep Disorders criteria (27). Individuals with concomitant OSA were only included if the two conditions were unequivocally distinguishable by polysomnography (PSG). In uncertain cases, the diagnosis of RBD was either confirmed by repeat PSG with the use of continuous positive airway pressure (CPAP), or the individuals were excluded from the study. Patients taking antidepressants at the time of v-PSG were not excluded from the study, if the opinion of their sleep specialist was that the onset of RBD symptoms was not temporally related to commencing SSRI medications. The selection of participants with RBD within the Oxford Discovery Cohort has been covered by Barber et al. (28).

### Cognitive assessment

To minimize fatigue, the cognitive tasks were split into two batteries intended for completion on consecutive days. Each battery, taking approximately 40 min, included a motor control task, a short questionnaire (for study ID, group, medication, symptom intensity), and a subset of the cognitive tasks. The tasks assessed attention (Target Detection), reaction time (SRT), memory (immediate and delayed word recognition), working memory (Digit Span, Spatial Span, Paired Associate Learning, Card Pairs), visuospatial processing (2D Manipulations, Four Towers/3D Scene Rotation, Picture Completion), emotion discrimination, spatial planning (Blocks, Tower of London), cognitive control (Switching Stroop, Trail Making), semantic reasoning (Verbal Analogies), and crystallized intelligence (Word Definitions). Detailed descriptions are available in Supplementary Tables S1, S2, and Supplementary Task Descriptions.

### Metacognitive assessment

In response to the question “How well do you think you performed on the task?” participants assessed their performance on each task using a 0–100 confidence judgment (CJ) slider, with 0 indicating no confidence (“poor”) and 100 indicating complete confidence (“excellent”) in their task performance.

### Statistical analysis

Task performance was standardized to a 0–100 percentage of maximum possible (POMP) scale, matching CJ scores (25), then metacognitive accuracy was determined by subtracting actual task performance from CJ scores for each task. No difference indicated perfect metacognitive accuracy, whereas differences closer to +100 suggest overestimation (positive bias), while those nearing −100 indicate underestimation (negative bias) of cognitive ability.

Prior to analysis, task performance scores were adjusted to the effects of age by decade, sex and years of further education using a linear regression model. Afterwards they were rank inverse transformed. ANOVA analyses were run to determine effects of task, groups, nuisance variables, and their interactions on POMP scores, CJ scores and metacognitive accuracy. Tukey post-hoc tests were run to identify which group differences drove main effects. A factor analysis with one factor was used to define the global cognitive composite, CJ composite and metacognitive accuracy composite score. Finally, simple Pearson correlations were run to assess the strength of the relationship between numerical variables. All statistical analyses were run in python using the statsmodels package (29).

## Results

### Sociodemographic and clinical characteristics

Demographics of participants are reported in Table 1. There was a difference in mean ages at the assessment, with controls being on average half a decade older than the patient population.

**Table 1:** Clinical and demographic characteristics of participants at the visit closest to the cognitive assessment.

	Controls	PD	RBD	Significant differences
Age at cognitive assessment	72.84 ± 8.37	66.01 ± 8.59	68.67 ± 8.78	***
Age at clinical assessment	71.66 ± 8.6	65.24 ± 8.79	68.09 ± 8.66	***
Hoehn & Yahr	N/A	2 ± 0.47	0.06 ± 0.31	***
Probability of idiopathic PD	N/A	95.36 ± 5.67	N/A	N/A
Epworth sleepiness scale	5.53 ± 3.2	7.66 ± 4.24	5.61 ± 3.89	*
REM sleep behavioral disorder screening questionnaire	2.15 ± 1.77	5.04 ± 3.14	9.45 ± 2.48	***
Age at PD/RBD diagnosis	N/A	56.95 ± 9.09	63.23 ± 8.63	***
Age at motoric symptom	N/A	55.18 ± 9.4	58.83 ± 9.98	ns
Disease duration since motoric symptom onset in years at the latest clinical visit	N/A	10.06 ± 2.59	N/A	N/A
Disease duration since diagnosis in years at the latest clinical visit	N/A	8.329 ± 1.9	4.86 ± 3.31	N/A

Means ± SD are presented for all scores, for all participants who took part in the study: *N* = 50 Healthy Controls (42% female; 4.95 ± 2.34 years of further education), *N* = 59PD patients (48% female; 4.2 ± 2.77 years of further education), *N* = 54 RBD patients (11% female; 4.35 ± 2.5 years of further education). There was no significant difference between participants years of further education. Participant ethnicity was 98% white background. ANOVA and t-tests results significance: * for *p* < 0.05; ** for *p* < 0.01; *** for *p* < 0.001. N/A indicates where data were not collected.

### Differences in retrospective CJ and metacognitive accuracy

The analysis pertaining to differences in cognitive performance across patient groups has been reported elsewhere (24). Compared to control participants, patients with PD underperformed on several tasks: Target Detection, Immediate Recognition Memory, Switching Stroop, Word Definitions, Blocks, Simple Reaction Time (SRT), Trail Making, and Picture Completion. Individuals with RBD demonstrated underperformance on the Immediate Recognition Memory, Word Definitions, Verbal Analogies, and SRT tasks. Here, we assess patients’ retrospective CJ, as well as their metacognitive accuracy, defined as the difference between the CJ and POMP.

**Figure 1:**
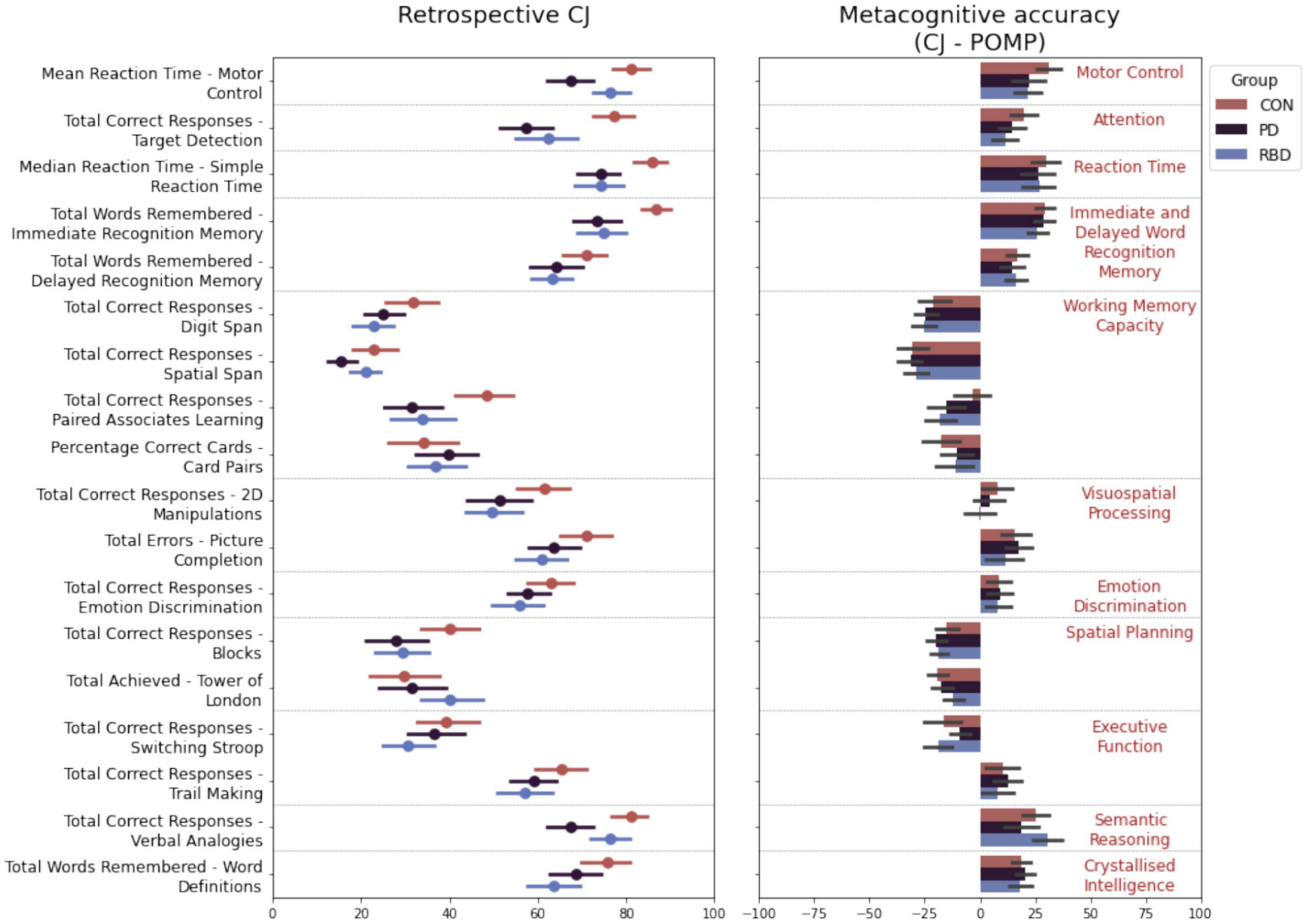
Differences between participants with PD, RBD, and healthy controls in confidence in task performance and metacognitive accuracy. Error bars represent the 95 confidence interval.

Repeated measures ANOVA analyses revealed significant differences between groups and their interaction with tasks on CJ, with a significant main effect of group [*F*(2, 2,845) = 41.35, *p* < 0.001] and a significant interaction [*F*(34, 2,845) = 1.77, *p* < 0.001]. In general, cross group differences in CJ aligned with where the patients showed cognitive performance deficits relative to the controls (Figure 1).

PD patients perceived their performance as worse than controls on tasks they underperformed on: Target Detection (meandiff = −19.06; *p* < 0.001), Immediate Recognition Memory (meandiff = −13.17; *p* = 0.001), Blocks (meandiff = −12.60; *p* = 0.02), and SRT (meandiff = −11.01; *p* = 0.003) but not Switching Stroop, Word Definitions and Picture Completion. Additionally, they perceived deficits in the Verbal Analogies (meandiff = −12.8539; *p* = 0.0008), Spatial Span (meandiff = −7.47; *p* = 0.02), Pairs Associate Learning (meandiff = −13.17; *p* = 0.001), and the Motor Control Task (meandiff = −12.85; *p* < 0.0001).

RBD patients perceived their performance as worse on tasks they underperformed on: Immediate Recognition Memory (meandiff = −11.74; *p* = 0.005), Word Definitions (meandiff = −11.88; *p* = 0.01), and SRT (meandiff = −11.28; *p* = 0.003), but not Verbal Analogies. Additionally, they perceived their performance as lower than controls on the Digit Span Task (meandiff = −8.71; *p* = 0.04) Pairs Associate Learning (meandiff = −11.74; *p* = 0.005), and Picture Completion (meandiff = −9.95; *p* = 0.04), but better than PD on Motor Control (meandiff = 8.57; *p* = 0.03).

T-tests against the null hypothesis revealed that with the exception of RBD patients on 2D manipulations and Trail Making, PD patients on 2D manipulations and control participants on Emotional Discrimination and Pairs Associate Learning, all groups had improper metacognitive accuracy (positive bias – closer to 100; or negative bias – closer to −100) on all tasks. A pattern was evident where participants across all groups generally underestimated their performance (negative bias) on tasks that assess working memory capacity and spatial planning, but overestimated their performance (positive bias) on tasks assessing motor control, reaction time, attention, word recognition, visuospatial processing, semantic reasoning and crystallized intelligence.

Where cross-group differences in cognition and CJ were noted, metacognitive accuracy differences were not. For metacognitive accuracy, the main effect of group [*F*(2, 2,843) = 2.00, *p* = 0.14] or the interaction between group and task [*F*(34, 2,843) = 1.33, *p* = 0.10] were not significant, but there was a significant effect of task [*F*(17,2,843) = 112.04, *p* < 0.001]. Group differences in metacognitive accuracy were only observed for the Paired Associates Learning task where the RBD group had a higher positive bias relative to the CON group (meandiff = 15.22; *p* = 0.02); however this did not survive correction for multiple comparisons.

**Figure 2:**
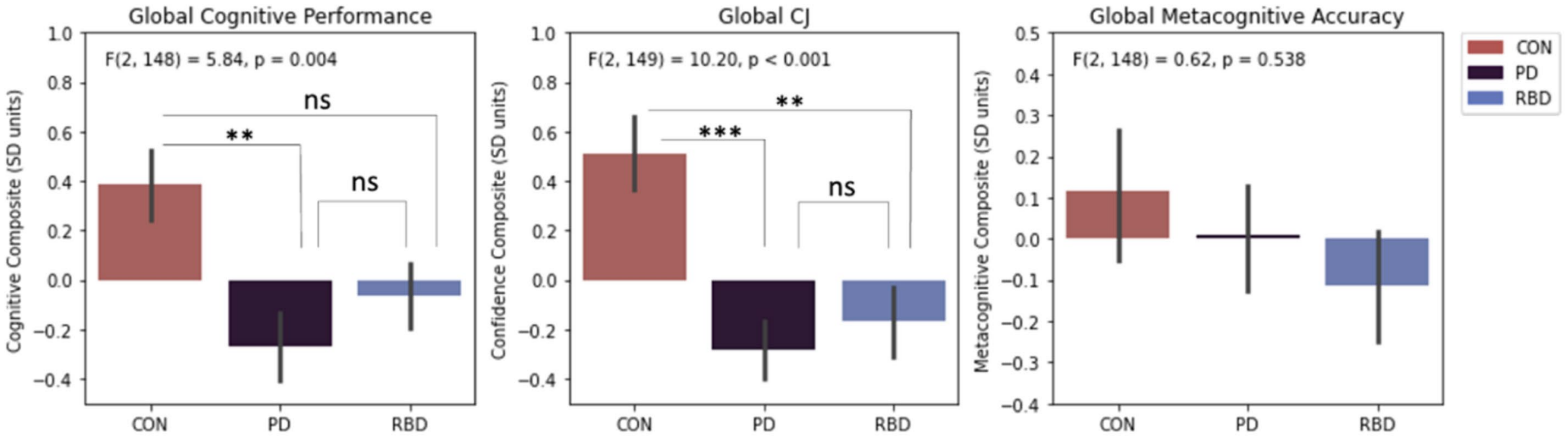
Differences in composite cognitive performance, CJ and metacognitive accuracy. Error bars represent standard error of the mean. Results significance: ns, not significant, * for *p* < 0.05; ** for *p* < 0.01; *** for *p* < 0.001.

One-way ANOVA analyses indicated significant group mean differences in global cognitive performance and CJ, but not metacognitive accuracy. Tukey post-hoc tests revealed PD patients had worse global cognitive performance than controls, whereas RBD patients did not. Both PD and RBD patients had worse global CJ than controls. PD and RBD patients were not different to each other in terms of global cognitive performance or global CJ (Figure 2).

### Differences in anxiety, depression, and MDS-UPDRS assessments

**Figure 3:**
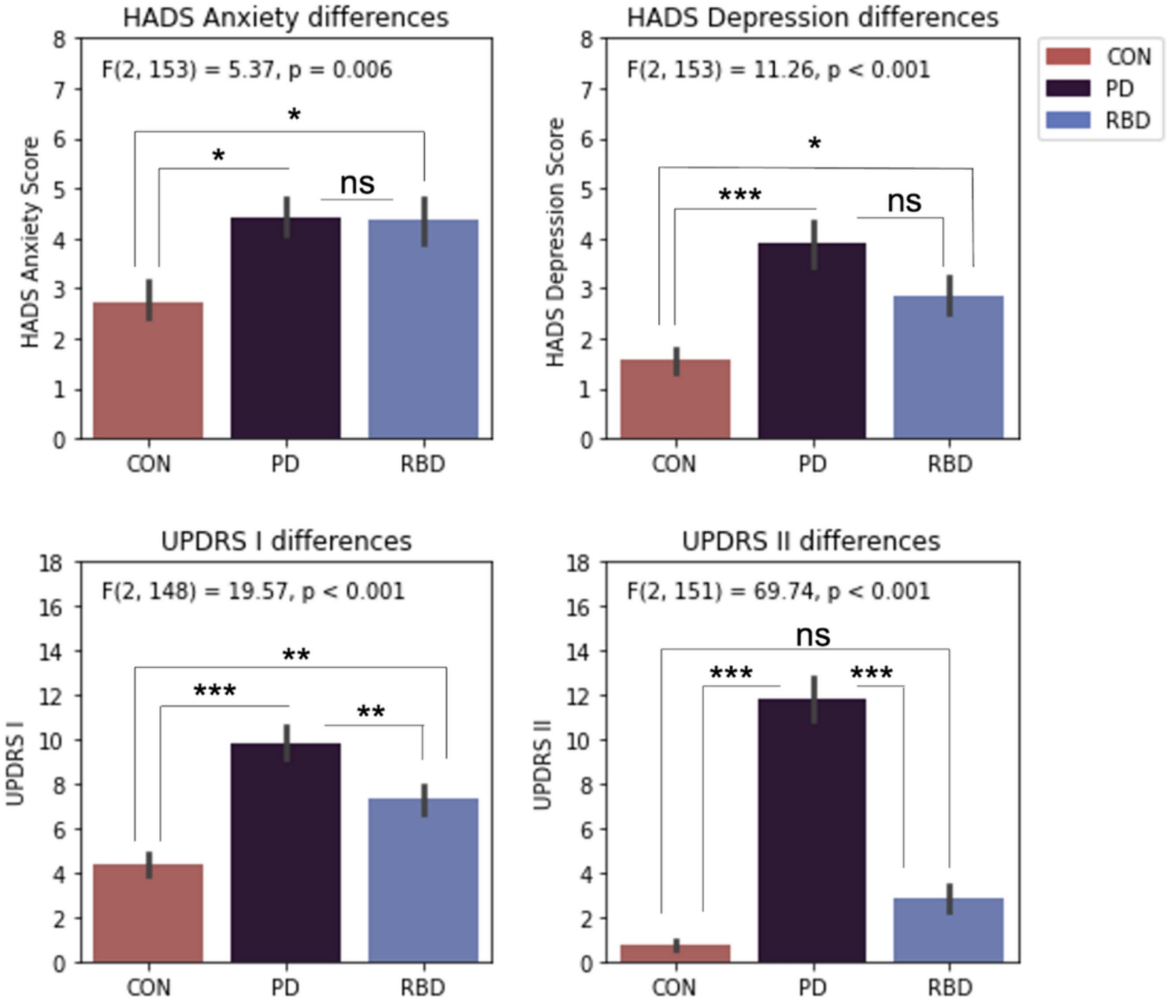
Differences in HADS and MDS-UPDRS scales. Error bars represent standard error of the mean. Results significance: ns, not significant, * for *p* < 0.05; ** for *p* < 0.01; *** for *p* < 0.001.

The average group scores on the HADS anxiety and HADS depression subscales were at subclinical levels. One way ANOVA analyses indicated significant mean group differences for anxiety, depression, MDS-UPDRS I and MDS UPDRS II. Tukey post-hoc tests revealed both patient groups had significantly worse anxiety and depression than control participants, but they were not significantly different to each other (Figure 3).

Only PD and RBD patients completed the MDS-UPDRS III scale. T-tests revealed significantly different scores, with PD patients having an average score of 30.67 ± 9.87, and RBD patients having an average score of 6.83 ± 6.97.

### Relationship between task performance, CJ, metacognitive accuracy, and clinical metrics

**Figure 4:**
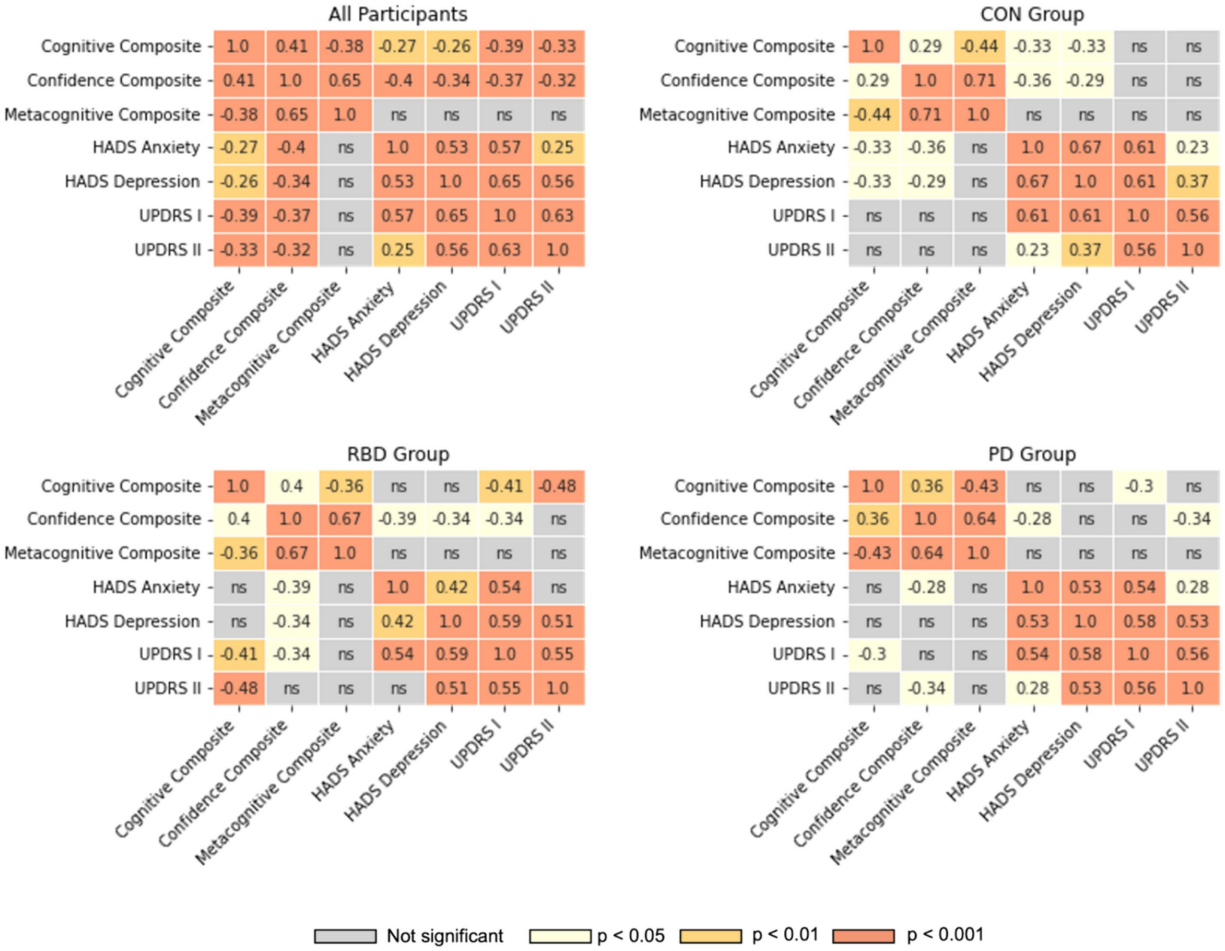
Correlation heatmaps between cognitive performance, confidence, metacognitive accuracy, HADS and MDS-UPDRS scales. Pearson correlation coefficients are illustrated for all significant correlations; ns, not significant.

All mental health and clinical symptoms metrics had medium-strength correlations with one another (Figure 4). There were no significant relationships between metacognitive accuracy and symptoms of anxiety or depression, or the MDS-UPDRS scales. The RBD group displayed a weak to medium inverse correlation between depression, anxiety, the MDS-UPDRS I scale and their confidence in their cognitive performance. The PD group displayed a weak to medium inverse correlation between anxiety, MDS-UPDRS II scale and their confidence in cognitive performance. In control participants a weak to medium inverse correlation was only observed between anxiety, depression and their confidence in cognitive performance, but not either MDS-UPDRS scales. For the PD group additional correlations were run with the MDS-UPDRS-III scale, but were not significant.

### Additional modeling of interaction effects on metacognitive accuracy

Additional linear models were run to investigate interaction effects of group membership with anxiety, depression and global cognitive performance on global metacognitive accuracy. Those revealed no significant main or interaction effects of group classification, HADS anxiety and depression scores, or MDS-UPDRS I and II scores on metacognitive accuracy (Supplementary material—Additional modeling). However, global cognitive performance had a significant main effect on metacognitive accuracy [*F*(1, 145) = 29.09, *p* < 0.001], though its interaction with group classification was not significant.

## Discussion

We identified no substantial difference in metacognitive accuracy in PD and RBD patients compared to controls. There was an inverse relationship between global cognitive performance and metacognitive accuracy, suggesting an increased positive bias associated with lower cognitive performance. Patients displayed lower confidence in their performance in tasks where cognitive deficits were noted relative to controls, but also additional domains where performance was preserved. Moreover, there was a significant inverse correlation between confidence and symptoms of depression and anxiety, as well as motor and non-motor difficulties encountered in daily living. This underscores the potential influence of emotional and functional challenges on the self-evaluation of cognitive capabilities in these patients. We contextualize these findings with the literature below.

Across all groups, we observed a slight negative bias in domains such as working memory and spatial planning, contrasted with a slight positive bias in areas including motor control, reaction time, and attention, as well as in word recognition, visuospatial processing, semantic reasoning, and crystallized intelligence. The nature of the task design and the manner in which feedback was being provided could have influenced these results by affecting participants’ CJ (from which standardized cognitive performance was subtracted). For example, immediate negative feedback on tasks such as the working memory tasks could have led to lower confidence, whereas tasks with reward accumulation as in the case of attention tasks could have led to higher confidence in performance. Performance in different cognitive domains might have also been regarded as more difficult than in others. Additionally, the older age of participants might have impacted their confidence in the cognitive performance more generally and independently of task specific factors. However, the biases in metacognitive accuracy were generally low. Calibrating a model to infer absolute metacognitive accuracy was beyond the scope of the study, and would require a large normative dataset.

We found a significant main effect of global cognitive performance on metacognitive accuracy. Our data further indicated an inverse correlation between these two variables, which was not group specific. This observation aligns with existing literature (17), which posits that patients with more advanced pathological conditions characterized by worse cognitive performance might lack awareness of their state. This also leads to the inference that significant deficits in metacognitive accuracy may not manifest in the early stages of the condition.

We discerned no significant difference in metacognitive accuracy of early to mid-stage PD and RBD patients compared to controls. This observation, however, does not preclude the emergence of substantial impairments at more advanced stages. Our focus was on individuals with MoCA scores above 24, specifically targeting those at or above the threshold for mild cognitive impairment (30), thus excluding more pronounced cognitive deficits. Therefore the results highlight that in the early stages of their condition, PD and RBD patients maintain a high level of awareness regarding their cognitive status. Such awareness resonates with patients’ expressed need for more research into cognitive deficits (10), and aligns with findings suggesting preserved self-awareness in PD (6). PD patients, in particular, often exhibit a keen understanding of their pathology and symptom progression (31). Therefore, the absence of detected metacognitive deficits in our study is likely reflective of the actual state of cognitive self-awareness in the early stages of these conditions rather than the methodology not being powered to detect changes. Employing the same analytical techniques and technology platform, previously revealed significant metacognitive impairments in moderate to severe traumatic brain injury patients (25).

Previously, we observed distinct cognitive profiles in PD and RBD patients, with both groups exhibiting cognitive deficits in task performance accuracy relative to the control population, yet PD patients additionally faced challenges in reaction time related to motor speed (24). In tasks where cognitive performance was lower than controls, both PD and RBD groups displayed reduced confidence. In PD, alignment was seen between confidence and performance in attention, recognition memory, visuospatial abilities, executive function, and crystallized intelligence. However, these patients also exhibited low confidence in language, working memory, and motor control. For RBD patients, while there was alignment in recognition memory, crystallized intelligence, and reaction time, they also showed decreased confidence in working memory capacity and visuospatial processing.

Lower confidence in motor control tasks among PD patients aligns with expectations given the prominent motor symptoms in PD, even at early stages where these difficulties might not be as pronounced (32). In RBD, the known progressive worsening of visuospatial cognitive deficits (33) could account for the notably lower confidence, possibly reflecting early patient awareness of their cognitive status, even when deficits are not yet distinctly measurable at a group level. Both patient groups displayed lower performance relative to the control population and reduced confidence in immediate recognition memory, and interestingly, poorer confidence in working memory capacity, despite no evident differences relative to the control population in this domain. This might suggest a sensitivity to task types used in assessing specific memory nuances in these pathologies, a factor noted to contribute to discrepant observations in memory task performance, confidence, and metacognition in PD (34). Galarneau (35) also highlights that self-ratings of memory in PD might be unreliable and require contrasting with objective assessments for accuracy. Finally, both PD and RBD patients exhibited globally lower confidence than controls.

Depression, anxiety, motor and non-motor difficulties of daily living were found to be significantly correlated across groups. However, correlations between these metrics with cognitive performance, confidence in performance and metacognitive accuracy were characterized by group specificity. Patients exhibited higher levels of depression and anxiety symptoms than controls, alongside greater difficulties in motor and non-motor aspects of daily life. This prevalence of mental health challenges in PD patients is well-documented (36) and similarly observed in RBD populations (37). Mental health variables and functional issues were inversely correlated with global confidence in cognitive performance for both groups. For PD a notable inverse correlation was observed between anxiety, difficulties in motor aspects of daily living, and confidence. However, this reduced confidence is less likely a product of anxiety *per se* and more an indicator of the severity of ongoing PD-specific symptoms. This is consistent with findings that PD patients experiencing motor fluctuations often suffer from generalized anxiety disorder more frequently than those without such fluctuations. Furthermore, patients report that their anxiety symptoms do not always align temporally with specific motor states, although when a correlation exists, it is typically associated with ‘off’ periods when they experience more intense motor symptoms (38). For RBD patients anxiety, depression, and non-motor aspects of daily living also show an inverse correlation with confidence in cognitive performance. Notably, RBD is associated with a poorer quality of life (39), despite being in a prodromal phase of pathology. Taken together, our findings serve as evidence that there is a significant level of awareness among these patients regarding their symptoms and cognitive status during the early stages of their condition.

While remote cognitive assessments may lack the control of clinical settings, we advised participants to minimize distractions during the assessment; yet strict adherence to this guidance could not be consistently verified, potentially affecting outcome reliability. However, our prior experience indicated a high level of participant compliance with remote assessments on this platform, as demonstrated in previous studies (25); (40). This was especially relevant for patients from our established cohort, who were accustomed to research protocols and thus more likely to adhere closely to them. Another limitation was the temporal lag between the administration of the HADS and MDS-UPDRS scales and the cognitive assessments, which might have led to an evolution in patients’ symptoms of depression, anxiety, and daily living difficulties over time.

Our findings indicate that the interplay between emotional state, difficulties with daily activities, and confidence in cognitive performance is pronounced in PD and RBD patients. This association necessitates further investigation, particularly in terms of controlling for potential confounding effects when measuring confidence and, implicitly, metacognitive accuracy in these patient populations. Moreover, examining PD patients with more advanced disease progression, including those with mild cognitive impairment or more significant deficits, could yield insightful data. Such an approach would allow exploration into whether cognitive and metacognitive differences relative to healthy individuals become more pronounced with the compounded effects of aging and PD progression. Equally, a simultaneous study of PD and RBD populations would be beneficial, to discern if patients initially diagnosed with RBD experience more significant metacognitive changes as their pathology progresses compared to those with PD alone, considering the known association of RBD with more severe cognitive deficits upon progression to PD. Furthermore, exploring other synucleinopathies in parallel (such as Lewy body dementia and multiple systems atrophy) with the same methodology could yield additional insights with respect to the preservation of metacognitive abilities across diverse patient groups. The inclusion of CJ in cognitive studies is both feasible and valuable, as they are not time-consuming and provide a deeper understanding of patients’ self-perceived cognitive abilities. This method could be pivotal in longitudinally tracking cognitive changes, thereby contributing to a more comprehensive patient care approach and a better understanding of disease trajectories in PD and RBD. Ultimately, our study underscores the importance of holistic patient care that takes into account patients’ awareness of their own condition and implicitly their ability to self-report symptoms to clinicians, as well as their psychoemotional states and how these correlate with their symptoms and experiences of daily living.

## Data availability statement

The datasets presented in this article will be made available upon reasonable request. Requests to access the datasets should be directed to AH, a.hampshire@imperial.ac.uk.

## Ethics statement

Ethical approval was given by the South Central-Oxford A Research Ethics Committee in accordance with the Declaration of Helsinki 1964, Ethics Ref: 16/SC/0108. The studies were conducted in accordance with the local legislation and institutional requirements. The participants provided their written informed consent (via email) to participate in this study.

## Author contributions

MB: Conceptualization, Data curation, Formal analysis, Investigation, Methodology, Visualization, Writing – original draft, Writing – review & editing, Project administration. FA: Data curation, Project administration, Writing – review & editing. NB: Methodology, Writing – review & editing. JW: Data curation, Project administration, Writing – review & editing. JR: Data curation, Project administration, Writing – review & editing. PM: Writing – review & editing. MH: Conceptualization, Funding acquisition, Project administration, Resources, Supervision, Writing – review & editing. AH: Supervision, Writing – review & editing.

## Footnotes

^1^ https://www.cognitron.co.uk

## References

[1] Brown A. Metacognition, executive control, self-regulation, and other more mysterious mechanisms. (eds.) F. Weinert, and R. Kluwe, Metacogn Motivat Understand. (1987). Erlbaum, Hillsdale, NJ 65–116.

[2] Fleming SM Dolan RJ. The neural basis of metacognitive ability. Philos Trans R Soc B. (2012) 367:1338–49. doi: 10.1098/rstb.2011.0417, PMID: 22492751 PMC3318765

[3] Seow TXF Rouault M Gillan CM Fleming SM. How local and global metacognition shape mental health. Biol Psychiatry. (2021) 90:436–46. doi: 10.1016/j.biopsych.2021.05.013, PMID: 34334187

[4] Jang Y Lee H Kim Y Min K. The relationship between metacognitive ability and metacognitive accuracy. Metacogn Learn. (2020) 15:411–34. doi: 10.1007/s11409-020-09232-w

[5] Palmer EC David AS Fleming SM. Effects of age on metacognitive efficiency. Conscious Cogn. (2014) 28:151–60. doi: 10.1016/j.concog.2014.06.007, PMID: 25064692 PMC4154452

[6] Sunderaraman P Cosentino S. Integrating the constructs of Anosognosia and Metacognition: a review of recent findings in Dementia. Curr Neurol Neurosci Rep. (2017) 17:27. doi: 10.1007/s11910-017-0734-1 28283961 PMC5650061

[7] Fleming SM Ryu J Golfinos JG Blackmon KE. Domain-specific impairment in metacognitive accuracy following anterior prefrontal lesions. Brain. (2014) 137:2811–22. doi: 10.1093/brain/awu221, PMID: 25100039 PMC4163038

[8] Karagulle Kendi AT Lehericy S Luciana M Ugurbil K Tuite P. Altered diffusion in the frontal lobe in Parkinson disease. AJNR Am J Neuroradiol. (2008) 29:501–5. doi: 10.3174/ajnr.A0850, PMID: 18202242 PMC8118887

[9] Kehagia AA Barker RA Robbins TW. Cognitive impairment in Parkinson’s disease: the dual syndrome hypothesis. Neurodegener Dis. (2013) 11:79–92. doi: 10.1159/000341998, PMID: 23038420 PMC5079071

[10] Schipper K Dauwerse L Hendrikx A Leedekerken JW Abma TA. Living with Parkinson’s disease: priorities for research suggested by patients. Parkinsonism Relat Disord. (2014) 20:862–6. doi: 10.1016/j.parkreldis.2014.04.025, PMID: 24874526

[11] Severiano E Sousa C Alarcão J Pavão Martins I Ferreira JJ. Frequency of dementia in Parkinson’s disease: a systematic review and meta-analysis. J Neurol Sci. (2022) 432:120077. doi: 10.1016/j.jns.2021.120077 34896923

[12] Thanvi BR Munshi SK Vijaykumar N Lo TCN. Neuropsychiatric non-motor aspects of Parkinson’s disease. Postgrad Med J. (2003) 79:561–5. doi: 10.1136/pmj.79.936.561 14612597 PMC1742855

[13] Haba-Rubio J Frauscher B Marques-Vidal P Toriel J Tobback N Andries D. Prevalence and determinants of rapid eye movement sleep behavior disorder in the general population. Sleep. (2018) 41:197. doi: 10.1093/sleep/zsx197, PMID: 29216391

[14] Postuma RB Iranzo A Hu M Högl B Boeve BF Manni R. Risk and predictors of dementia and parkinsonism in idiopathic REM sleep behaviour disorder: a multicentre study. Brain. (2019) 142:744–59. doi: 10.1093/brain/awz030, PMID: 30789229 PMC6391615

[15] Postuma RB Gagnon J-F Bertrand J-A Génier Marchand D Montplaisir JY. Parkinson risk in idiopathic REM sleep behavior disorder: preparing for neuroprotective trials. Neurology. (2015) 84:1104–13. doi: 10.1212/WNL.0000000000001364 25681454 PMC4371408

[16] Oh-Lee JD Szymkowicz SM Smith SL Otani H. Metacognitive performance, the tip-of-tongue experience, is not disrupted in parkinsonian patients. Parkinson’s Dis. (2012) 2012:1–12. doi: 10.1155/2012/174079, PMID: 22577598 PMC3347746

[17] Orfei MD Assogna F Pellicano C Pontieri FE Caltagirone C Pierantozzi M. Anosognosia for cognitive and behavioral symptoms in Parkinson’s disease with mild dementia and mild cognitive impairment: frequency and neuropsychological/neuropsychiatric correlates. Parkinsonism Relat Disord. (2018) 54:62–7. doi: 10.1016/j.parkreldis.2018.04.015, PMID: 29709507

[18] White TL Sadikot AF Djordjevic J. Metacognitive knowledge of olfactory dysfunction in Parkinson’s disease. Brain Cogn. (2016) 104:1–6. doi: 10.1016/j.bandc.2016.01.004 26867087

[19] Mack J Okai D Brown RG Askey-Jones S Chaudhuri KR Martin A. The role of self-awareness and cognitive dysfunction in Parkinson’s disease with and without impulse-control disorder. JNP. (2013) 25:141–9. doi: 10.1176/appi.neuropsych.12030076, PMID: 23686032

[20] Angioletti L Campanella S Balconi M. Metacognition deficits and impulsivity in Parkinson’s disease patients with and without gambling behavior: a pilot study. Neurol Psychiatry Brain Res. (2020) 36:88–95. doi: 10.1016/j.npbr.2020.04.002

[21] Fernie BA Spada MM Ray Chaudhuri K Klingelhoefer L Brown RG. Thinking about motor fluctuations: an examination of metacognitions in Parkinson’s disease. J Psychosom Res. (2015) 79:669–73. doi: 10.1016/j.jpsychores.2015.05.001, PMID: 25990617

[22] Allott R Wells A Morrison AP Walker R. Distress in Parkinson’s disease: contributions of disease factors and metacognitive style. Br J Psychiatry. (2005) 187:182–3. doi: 10.1192/bjp.187.2.182, PMID: 16055832

[23] Szewczyk-Krolikowski K Tomlinson P Nithi K Wade-Martins R Talbot K Ben-Shlomo Y. The influence of age and gender on motor and non-motor features of early Parkinson’s disease: initial findings from the Oxford Parkinson disease center (OPDC) discovery cohort. Parkinsonism Relat Disord. (2014) 20:99–105. doi: 10.1016/j.parkreldis.2013.09.025, PMID: 24183678

[24] Bălăeţ M Alhajraf F Zerenner T Welch J Razzaque J Lo C. Online cognitive monitoring technology for people with Parkinson’s disease and REM sleep behavioural disorder. NPJ Digit Med. (2024) 7:118. doi: 10.1038/s41746-024-01124-6, PMID: 38714742 PMC11076465

[25] Bourke NJ Trender W Hampshire A Lai H Demarchi C David M. Assessing prospective and retrospective metacognitive accuracy following traumatic brain injury remotely across cognitive domains. Neuropsychol Rehabil. (2023) 33:574–91. doi: 10.1080/09602011.2022.2034650, PMID: 35168480

[26] Rizzo G Copetti M Arcuti S Martino D Fontana A Logroscino G. Accuracy of clinical diagnosis of Parkinson disease: a systematic review and meta-analysis. Neurology. (2016) 86:566–76. doi: 10.1212/WNL.0000000000002350 26764028

[27] American Academy of Sleep Medicine. International classification of sleep disorders. 3rd ed. Darien, IL: American Academy of Sleep Medicine (2014).

[28] Barber TR Lawton M Rolinski M Evetts S Baig F Ruffmann C. Prodromal parkinsonism and neurodegenerative risk stratification in REM sleep behavior disorder. Sleep. (2017) 40:71. doi: 10.1093/sleep/zsx071, PMID: 28472425 PMC5806544

[29] Seabold S Perktold J. Statsmodels: Econometric and statistical modeling with Python. 9th Python in Science Conference, Austin, Texas (2010). p. 92–96.

[30] Goldstein FC Ashley AV Miller E Alexeeva O Zanders L King V. Validity of the Montreal cognitive assessment as a screen for mild cognitive impairment and dementia in African Americans. J Geriatr Psychiatry Neurol. (2014) 27:199–203. doi: 10.1177/0891988714524630, PMID: 24614202

[31] Salinas MR Chambers EJ Ho T Khemani P Olson DM Stutzman S. Patient perceptions and knowledge of Parkinson’s disease and treatment (KnowPD). Clin Parkinsonism Related Disord. (2020) 3:100038. doi: 10.1016/j.prdoa.2020.100038, PMID: 34316624 PMC8298769

[32] Fahn S. Description of Parkinson’s disease as a clinical syndrome. Ann N Y Acad Sci. (2003) 991:1–14. doi: 10.1111/j.1749-6632.2003.tb07458.x 12846969

[33] Fantini ML Farini E Ortelli P Zucconi M Manconi M Cappa S. Longitudinal study of cognitive function in idiopathic REM sleep behavior disorder. Sleep. (2011). doi: 10.1093/sleep/34.5.619 PMC3079941 21532955

[34] Smith SJ Souchay C Moulin CJA. Metamemory and prospective memory in Parkinson’s disease. Neuropsychology. (2011) 25:734–40. doi: 10.1037/a0025475, PMID: 21928905

[35] Bégin Galarneau M-È. Self-ratings of memory in Parkinson’s disease: relation to depressive symptoms. Pers Exec Funct. (2019). doi: 10.20381/RUOR-23455

[36] Kuopio AM Marttila RJ Helenius H Toivonen M Rinne UK. The quality of life in Parkinson’s disease. Movement Disord. (2000) 15:216–23. doi: 10.1002/1531-8257(200003)15:2<216::AID-MDS1003>3.0.CO;2-# 10752569

[37] Lam SP Zhang J Tsoh J Li SX Ho CKW Mok V REM Sleep Behavior Disorder in Psychiatric Populations. J Clin Psychiatry (2010) 71:1101–1103. doi: 10.4088/JCP.l05877gry 20797385

[38] Leentjens AFG Dujardin K Marsh L Martinez-Martin P Richard IH Starkstein SE. Anxiety and motor fluctuations in Parkinson’s disease: a cross-sectional observational study. Parkinsonism Relat Disord. (2012) 18:1084–8. doi: 10.1016/j.parkreldis.2012.06.007, PMID: 22771284

[39] Kim KT Motamedi GK Cho YW. Quality of life in patients with an idiopathic rapid eye movement sleep behaviour disorder in Korea. J Sleep Res. (2017) 26:422–7. doi: 10.1111/jsr.12486, PMID: 28019055

[40] Del Giovane M Trender WR Bălăeţ M Mallas E-J Jolly AE Bourke NJ, Computerised cognitive assessment in patients with traumatic brain injury: an observational study of feasibility and sensitivity relative to established clinical scales. eClinicalMedicine (2023) 59:101980. doi: 10.1016/j.eclinm.2023.101980 37152359 PMC10154960

