# Supplementary material for: Metacognitive accuracy differences in Parkinson’s disease and REM sleep behavioral disorder relative to healthy controls
Source: Front Neurol. 2024 May 27;15:1399313. doi: 10.3389/fneur.2024.1399313 (PMC11164050; doi:10.3389/fneur.2024.1399313)
Supplement: Supplementary file 1 [file Presentation_1.pdf]

SUPPLEMENTARY MATERIALS

Metacognitive accuracy differences in Parkinson's Disease and REM Sleep Behavioural Disorder relative to healthy controls

Battery 1 (Day 1)	Operational complexity	*Battery 2 (Day 2)*	Operational complexity	Notes
Motor control	1	Motor control	1	
Recognition memory immediate	1	SRT	1	
Target detection	1	Trail making	1	
Emotional discrimination	1	Paired associate learning	2	
2D manipulations	1	Switching Stroop	2	
Digit Span	2	Picture completion	3	
Spatial Span	2	Card Pairs	3	
Blocks	3	3D Scene rotation (faulty towers)	3	Metacognitive accuracy was not assessed for this task
Tower of London	3			
Verbal Analogies	3			
Word definitions	3			
Recognition memory delayed	2			

Supplementary Table 1. Batteries administered to participants. Operational complexity: grade 1 - responses are simply tapping on the screen in response to stimuli, grade 2 - multiple taps are required for a single response, grade 3 - more complex reasoning and rule processing is required. This table has been reported previously in Balaet et al., 2024.

TASK DESCRIPTIONS - reproduced from Balaet et al., 2024

Motor control
This task is designed to measure motor control - which is defined as the ability to click on discrete stimuli on the screen quickly and with high accuracy. Participants must click on a number of targets that appear at random points on the screen. The mean reaction time, as well as the mean (euclidean) distance from the target are measured.

Recognition memory (immediate and delayed)
The test measures recognition memory for words at different time delays. The participant is presented with a sequence of objects in a pseudo-random order, each presented for 1000 ms and with an inter-image interval of 200 ms. The participant is then immediately presented with a sequence of 24 objects. 12 of the objects are the ones they were asked to commit to memory and 12 are new words, six of which are semantically similar foils to the ones committed to memory. At the end of the battery of tests, the participant’s recognition memory is probed again for the same target items alongside a different set of foils and distractors. The primary output for both immediate and delayed word memory is the sum of correct responses, with maximum score being 24.

Target detection
The Target Detection test measures spatial visual attention. The participant is presented with a target shape on the left of the screen and a probe area on the right side of the screen. After 3000ms, the probe area begins to fill with shapes, the participant must identify and click the target shape while ignoring the

distractor shapes. Shapes are added every 1000ms and a subset of the shapes in the probe area are removed every 1000ms. The trial runs for a total of 120 addition/removal cycles. The target shape is included in the added shapes pseudo randomly, at a frequency of 12 in 20 cycles. The primary output is the total number of target shapes clicked.

Emotional discrimination

This test measures an individual's ability to identify and discern between emotions. Participants are presented with pictures of two people, each expressing a particular emotion (e.g. happy, neutral, angry, scared). They must decide if the emotions expressed by each person are the same or different. Trials vary based on the emotions used as well as whether individuals have congruent vs. incongruent emotional expressions. To obtain maximum points, participants must complete 50 trials as accurately as possible. For every correct answer, the total score increases by one point. The outcome measure is the total score.

2D manipulations

The 2D Mental Manipulation test measures the ability to spatially manipulate objects in mind (Silverman et al., 2000). In this version of the test, a grid with coloured squares is presented at the top of the screen, with a further four grids with coloured squares presented below (i.e. probe grids). One of the four grids is identical to the target grid above but is rotated by either 90, 180 or 270 degrees whilst the other grids differ by five squares. To obtain maximum points, the participant must indicate which of the four grids is identical to target, solving as many problems as possible within three minutes.

Digit Span

This task is a proxy for working memory. Participants must remember a string of digits that increment in length. Each time participants remember the full string, the next string displayed increments in length by one unit. The total amount of strings participants recalled fully is recorded.

Spatial Span

The Spatial Span test measures spatial short-term memory capacity. It is a variant on the classic Corsi Block Tapping Test (Corsi, 1972). The participant is presented with a 4 x 4 grid, onto which is displayed a sequence of squares in different positions in the grid. The participant must then click the squares in the order that they were highlighted. The difficulty is incremented using a ratchet system, every time a sequence is recalled correctly, the length of the subsequent sequence is incremented by one. The test is terminated when three consecutive mistakes are made on a particular sequence length. The primary output is the maximum sequence length correctly recalled. Minimum level = 2, maximum level = 16, ISI = 0ms, encoding time = 1500ms.

Blocks

Participants must remove blocks from one array until it matches a target array; blocks will fall under gravity. The Blocks task has been adapted from a common analogue neuropsychological test in which the subject has to match a shape using coloured blocks. This is thought to be a good measure of spatial visualisation skills. The current version also incorporates a measure of planning as you have to predict the shapes that will be created once a block is removed and gravity takes effect.

Tower of London

The Tower of London test measures spatial planning. It is a variant on the original Tower of London Test (Shallice, 1982). The participant is shown two sets of three prongs with coloured beads on them. The first set is the initial state and the second set is the target state. The participant must work out the lowest number of moves it would take to transition from the initial state to the target state. They must then input this number using an on-screen number pad. This differs from the original test in that the participant is not allowed to move the beads, all calculation and planning must be done in their head. This is to prevent correct answers being reached through iterative error correction. The test consists of 10 trials of variable difficulty. The difficulty is scaled using the number of beads and the convolutedness, defined as the number of moves that must be made that do not place a bead in its final target position. The outcome measure is the total number of correct trials.

Verbal Analogies

The Analogical Reasoning test measures semantic reasoning abilities. In this version of the test, participants are presented with two written relationships that they must decide have the same type of association or not (e.g. “Lion is to feline as cabbage is to vegetable”). Participants must indicate their decision by selecting the True or False buttons presented below the written analogies. Analogies are varied across semantic distance to modulate difficulty and association types switch throughout the sequence of trials. To obtain maximum points, participants must solve as many problems as possible within three minutes. For every correct response, the total score increases by one. For every incorrect response, the total score decreases by one. The outcome measure is the total score.

Word Definitions

In this test, individuals are assessed on their ability to identify the correct definitions of words. Participants are presented with a word accompanied by four descriptive statements. They must decide which of the four statements provides the correct definition of the word. Words vary based on their frequency of use in English written language, resulting in rare and commonly used words being presented. For each word, the participant has twenty seconds to choose a definition. To obtain maximum points, participants must answer 21 word-definitions correctly. For every correct response, the total score increases by one point. The outcome measure is the total score.

Simple reaction task

This task is the simplest task available for measuring reaction time. Participants must respond as quickly as they can to a stimulus that appears on the screen at different timepoints. What is measured is the total time it takes for the participant to press on the screen after the stimulus appeared.

Trail making

Trail Making is based on a classical pen and paper neuropsychological test. The first section, containing only numbers reflects the speed at which the participant is able to process a field of information and sort it accurately. The second section, in which there are both numbers and letters requires ‘attentional switching’, which is an important executive function. Subtracting the former from the latter provides a measure of the ‘switching cost’. What is measured is the time required by the participant to click on all the targets in the correct order.

Pairs associate learning

This task is a measure of visual working memory. Participants must remember a series of objects and their associated locations on a grid. What is measured is the total amount of correct trials.

Switching stroop

This task is a proxy for executive function, and is a routine cognitive test that is used to classify patients suffering with conditions such as traumatic brain injury. Participants must describe the colour of a central tile by choosing between two words reading "Red" and "Blue" and coloured either red or blue. The participant must pay attention to either the text of the word or the colour of the word at any given time. What is recorded is the total number of correct trials.

Picture completion

This task is assessing the ability of the participants to identify how patterns taken out of their original context fit back within the context. This taps into visual processing and pattern recognition abilities. Participants must correctly assign missing pieces from a picture. The pieces can be rotated through 90, 180 or 270 degrees. What is recorded is the total number of errors participants make while trying to piece the pictures back together.

Card pairs

This task is a measure of working memory. Participants are shown an array of face up cards. These cards are then placed face down and the participant must remember and identify the locations of pairs of cards. What is measured is the percentage of pairs of cards the participant correctly identified. Formerly, card pairs games used to be a popular computerised entertainment game back in the 90'.

Four towers (3D Scene rotation)

Four Towers/Faulty Towers is a 3D Perspective Rotation test that measures the ability to picture a 3D scene and rotate it in the mind’s eye. The participant is presented with an array comprising four

perspective images each containing three tower blocks. Three of the images show the same tower blocks arranged in the same way but viewed from different perspectives. One of the images is the odd one out as it differs either by the type of tower blocks that are present or the relative locations that they are placed in. The participant must identify the odd one out as quickly and accurately as they can. A total of 12 problems are presented and the score increments or decrements by 1 dependent on whether or not the participant correctly identifies the odd one out. The primary output is total score.

Task	Primary Measure	Definition of Primary Measure	Secondary Measure	Definition of Secondary Measure
Motor Control	Accuracy	Mean Euclidean distance from the target, assessing precision in motor responses.	Mean Reaction Time	Average time taken to respond to motor control stimuli, assessing speed and accuracy.
Immediate Recognition Memory	Total Correct Responses	Sum of correctly identified words immediately after presentation, indicating immediate memory recall.	Median Reaction Time	Median time to react to each stimulus in the immediate memory test.
Delayed Recognition Memory	Total Correct Responses	Sum of correctly identified words after a delay, reflecting delayed memory retention.	Median Reaction Time	Median time to react to each stimulus in the delayed memory test.
2D Manipulations	Total Correct Responses	Number of spatial problems correctly solved, indicating spatial manipulation ability.	Median Reaction Time	Median time to react to each spatial manipulation problem.
Emotion Discrimination	Total Correct Responses	Number of correctly identified emotions in images, measuring emotion recognition accuracy.	Median Reaction Time	Median time to react to each emotion discrimination trial.

Blocks	Total Correct Responses	Number of correctly matched block arrangements, assessing spatial visualization and planning skills.	Median Reaction Time	Median time to react to each block arrangement task.
Target Detection	Total Correct Responses	Total number of correctly identified target shapes, reflecting spatial attention and accuracy.	Mean Reaction Time	Average time to react to each target detection stimulus.
Spatial Span	Total Correct Responses	Longest sequence length correctly recalled in a spatial memory task.	Median Reaction Time	Median time to react during each spatial memory recall attempt.
Verbal Analogies	Total Correct Responses	Total number of correctly solved semantic analogies, indicating semantic reasoning ability.	Median Reaction Time	Median time to react to each analogy problem.
Word Definitions	Total Correct Responses	Number of correctly identified word definitions, measuring vocabulary knowledge and comprehension.	Median Reaction Time	Median time to react to each word definition choice.
Simple Reaction Time	Median Reaction Time	Median time taken to respond to a basic visual stimulus, measuring basic reaction speed.	-	-
Trail Making	Mean Reaction Time	Mean reaction time between letter and number reaction times in trail making tasks.	Mean Number Reaction Time	Average time to respond to number sequence tasks.

			Mean Letter Reaction Time	Average time to respond to letter sequence tasks.
			Number/Letter Cost	Additional time (cost) of switching between numbers and letters.
PAL (Paired Associates Learning)	Total Correct Responses	Number of correctly recalled object-location pairs, indicating visual working memory capacity.	Median Reaction Time	Median time to react during each object-location recall attempt.
Switching Stroop	Total Correct Responses	Total number of correctly completed trials in a Stroop task, assessing cognitive flexibility and inhibition.	Mean Incongruent Accuracy	Accuracy in identifying incongruent color-word matches.
			Median Reaction Time	Median time to react to each Stroop task stimulus.
			Mean Switch Accuracy	Accuracy in correctly identifying switches between color and text.
			Median Switch Reaction Time	Median time to react during switch tasks in the Stroop task.
			Median Incongruent Reaction Time	Median reaction time for identifying incongruent color-word matches.

Picture Completion	Total Errors	Number of errors made in picture completion tasks, assessing visual processing and pattern recognition.	Total Time Taken	Total duration taken to complete all picture completion tasks.
Digit Span	Total Correct Responses	Number of correctly remembered digit sequences, indicating working memory capacity.	Median Reaction Time	Median time to react during each digit recall attempt.
Tower of London	Total Correct Responses	Total number of correctly planned moves in a spatial planning task, assessing executive function and planning skills.	Median Reaction Time	Median time to react to each planning challenge in the task.
Four Towers	Total Correct Responses	Number of correctly identified unique towers in a 3D perspective rotation task, measuring spatial visualization ability.	Median Reaction Time	Median time to react to identify the unique tower.
Card Pairs	Percentage of Cards Correctly Identified	Percentage of card pairs correctly identified, assessing memory and attention to detail.	Median Array Time	Median time taken to identify pairs of cards in the memory task.

Table S2. Descriptions of tasks primary and secondary measures. This table has been previously reported by Balaet et al., 2024

Additional Modelling

Predicting metacognitive accuracy from group, HADS anxiety scores and their interaction revealed the group classification did not have a significant main effect ($F(2, 141) = 0.2$, $p = 0.81$), and neither did HADS anxiety scores ($F(1, 141) = 3.19$, $p = 0.08$) or the interaction between group and HADS anxiety scores ($F(2, 141) = 0.2$, $p = 0.82$). Similar findings were observed with HADS depression scores: no significant group effect on metacognitive accuracy ($F(2, 141) = 0.36$, $p = 0.70$), no significant main effect of HADS depression scores ($F(1, 141) = 0.62$, $p = 0.43$), and no significant interaction effect ($F(2, 141) = 0.18$, $p = 0.83$).

Predicting metacognitive accuracy from the group, MDS-UPDRS I score, and their interaction revealed no significant group effect ($F(2, 136) = 0.59$, $p = 0.55$), main effect of MDS-UPDRS I scores ($F(1, 136) = 0.08$, $p = 0.77$), or interaction effect ($F(2, 136) = 0.06$, $p = 0.94$). Similarly, no significant group effect ($F(2, 139) = 0.59$, $p = 0.55$), main effect of MDS-UPDRS II ($F(1, 139) = 0.15$, $p = 0.70$), or interaction effect ($F(2, 139) = 2.29$, $p = 0.10$) was observed.

Finally, predicting metacognitive accuracy from group, global cognitive performance and their interaction displayed a near-significant group effect on metacognitive accuracy ($F(2, 145) = 2.81$, $p = 0.06$) and a significant main effect of global cognitive performance ($F(1, 145) = 29.09$, $p < 0.001$). However, the interaction between group and cognitive performance was not significant ($F(2, 145) = 0.20$, $p = 0.82$).
